# Prevention and Management of Hormonal Crisis during Theragnosis with LU-DOTA-TATE in Neuroendocrine Tumors. A Systematic Review and Approach Proposal

**DOI:** 10.3390/jcm9072203

**Published:** 2020-07-12

**Authors:** Maria Isabel del Olmo-García, Maria Angustias Muros, Martín López-de-la-Torre, Marc Agudelo, Pilar Bello, Jose M. Soriano, Juan-Francisco Merino-Torres

**Affiliations:** 1 Endocrinology and Nutrition Department, University and Polytechnic Hospital La Fe, 46026 Valencia, Spain; merino_jfr@gva.es; 2 Nuclear Medicine Department, University Hospital Virgen de las Nieves, 18014 Granada, Spain; mangustias.muros.11pa@juntadeandalucia.es; 3 Endocrinology and Nutrition Department, University Hospital Virgen de las Nieves, 18014 Granada, Spain; m.endocrino@gmail.com; 4 Nuclear Medicine Department, University and Polytechnic Hospital La Fe, 46026 Valencia, Spain; marc-agudelo@hotmail.com (M.A.); bello_pil@gva.es (P.B.); 5 Food & Health Lab, Institute of Materials Science, University of Valencia, 46980 Valencia, Spain; jose.soriano@uv.es; 6 Joint Research Unit on Endocrinology, Nutrition and Clinical Dietetics, University of Valencia-Health Research Institute La Fe, 46026 Valencia, Spain

**Keywords:** neuroendocrine tumors, peptide receptor radionuclide therapy, 177Lu-DOTA-TATE, hormonal crisis, carcinoid syndrome, carcinoid crisis, catecholaminergic crisis, systematic review

## Abstract

Neuroendocrine tumors (NETs) frequently overexpress somatostatin receptors (SSTR) on their cell surface. The first-line pharmacological treatment for inoperable metastatic functioning well-differentiated NETs are somatostatin analogs. On second line, Lu-DOTA-TATE (^177^Lu-DOTA^0^ Tyr ^3^ octreotate) has shown stabilization of the disease and an increase in progression free survival, as well as effectiveness in controlling symptoms and increasing quality of life. The management of functional NETs before and during LU-DOTA-TATE treatment is specially challenging, as several complications such as severe carcinoid and catecholamine crisis have been described. The aim of this review is to establish practical guidance for the management and prevention of the most common hormonal crises during radionuclide treatment with Lu-DOTA-TATE: carcinoid syndrome (CS) and catecholamine hypersecretion, as well as to provide a brief commentary on other infrequent metabolic complications. To establish a practical approach, a systematic review was performed. This systematic review was developed according to the Preferred Reporting Items for Systematic Reviews and Meta-Analyses (PRISMA) statement and conducted using MEDLINE (accessed from PubMed), Google Scholar and ClinicalTrials.gov. Literature searches found 449 citations, and finally nine were considered for this systematic review.

## 1. Introduction

Neuroendocrine neoplasms (NENs) have been usually described as infrequent and rare tumors but their incidence has been rising over time [1]. Neuroendocrine cells are distributed widely throughout the body and these tumors may arise in most organs. NENs are classified according to the rate of proliferation (mitotic rate and Ki-67 proliferative index) and differentiation. Accordingly, the World Health Organization (WHO) has classified NENs as neuroendocrine tumors (NETs) subclassified as G1, G2 and G3 according to mitotic rate, proliferative index and poorly differentiated (neuroendocrine carcinomas NECs). This classification has been approved for gastrointestinal and pancreatic NENs. However, NENs arising in bronchial or other localizations do not follow this same classification [2].

Morphologically, well-differentiated NETs have characteristic organoid arrangements with glandular, trabecular or solid nest arrangements. These cells produce abundant neurosecretory granules as reflected in the strong and diffuse immunohistochemical expression of neuroendocrine markers. In addition, some tumors may secrete specific peptide hormones or bioamines which may produce clinically evident hormonal syndromes (functioning NETs) and their localization may vary (pancreas, midgut, foregut, bronchial) [3].

Paragangliomas and pheochromocytomas (PPGL) are another kind of rare neuroendocrine tumor with a reported annual incidence of 1 in 300.000. [4] PPGLs arise from the extra-adrenal autonomic paraganglia (paraganglioma) or adrenal medulla (pheochromocytoma). Paraganglia are small organs consisting mainly of neuroendocrine cells derived from the embryonic neural crest and may have the ability to secrete catecholamines (usually those on an infra-diafragmatic location) and therefore of producing hormonal syndromes as well as those which arise from the adrenal medulla (pheochromocytoma) [4].NETs are heterogeneous malignancies; however, many of them have in common their ability to overexpress somatostatin receptors (SSTR) on their cell surface. Five receptor subtypes for SSTR have been identified: SSTR1, SSTR2, SSTR3, SSTR4 and SSTR5. GEP-NENs (gastroenteropancreatic neuroendocrine neoplasms) especially overexpress SSTR2 compared to healthy tissue, which is the cornerstone of the concept of theragnosis in these tumors. The expression of SSTR may vary within the different NETs (Table 1) [5,6].

Among the nuclear medicine imaging techniques, some are based on SSTR expression, such as somatostatin receptor scintigraphy (SRS). Within the conventional radiopharmaceuticals used in SRS, 111In-labeled somatostatin analogs were initially developed: the best known is the 111In-labeled octreotide and diethylene triamine penta-acetic (DTPA), called 111In-DTPA -Pentreotide (111InOctreoscan^®^, Tyco Healthcare, Mallinckrodt, St Louis, MO, USA). For several years this has been considered the gold standard of functional imaging [6]. Subsequently, 99mTc-labeled radiopharmaceuticals which aim to use gamma-emitting isotopes, which are easier to handle and more available, were developed ((99mTc) such as EDDA-HYNIC-Thr3-octreotide ((99mTc) TEKTROTYD^®^, Polatom, Otwock, Poland) [7,8].

Positron emission tomography (PET) uses positron-emitting radioisotopes (18F, 11C, 68Ga), a new generation of tracers whose shorter half-life reduces patients’ radiation. In addition, PET offers a higher spatial resolution, detects more lesions, and allows a quantification of their uptake (standard uptake value, SUV) of great utility in therapy monitoring [9]. These are the studies currently considered of whenever they are available, since they change the follow-up strategy in more than 70% of patients [10].

Current recommendations suggest that the conventional 111In-pentetreotide single-photon emission computed tomography (SPECT/CT) scan should be replaced by the 68 Ga-DOTA-SSA PET/CT. 68 Ga-DOTA-SSA PET/CT has a greater sensitivity, shorter duration of image acquisition, better spatial resolution, diagnostic accuracy, lower radiation dose for the patient and [11] is more readily available in nuclear medicine departments (it is a generator product instead of a cyclotron). Its ability to penetrate tissues is higher, so that the radiation dose of 68 Ga-peptide is < 50% of the 111In-pentetreotide dose, and in addition 68 Ga-peptide has a higher affinity for the somatostatin receptor [12]. Basic research usually defines the specific molecular target of a specific disease (i.e., receptor, metabolite). Theragnosis involves changing the use of the radioisotope to offer the possibility of using molecular imaging beyond diagnostic purposes and developing targeted radionuclide therapy: One of the most widely used theragnostic agents targeting NETs is metaiodobenzylguanidine (MIBG), a guanethidine analogue of norepinephrine. 123I/131I-MIBG theragnostics have been applied in the clinical evaluation and management of NETs, especially in neuroblastoma, paraganglioma, and pheochromocytoma. 177Lu-DOTATE is also a theragnostic agent used in the field of NET treatment [13]. The first-line pharmacological treatment for inoperable metastatic functioning well-differentiated NETs are somatostatin analogs (SSA). SSA have proven antiproliferative effects in non-functional NETs as well as causing a substantial decrease in hormonal secretion and remission of hormonal hypersecretory syndromes. When disease progresses in this first line treatment other available medical treatments include everolimus, sunitinib, and cytotoxic chemotherapy. A promising targeted therapy is Peptide Receptor Radionuclide therapy (PRRT) with (Lutetium-177-DOTA0-Tyr3) octreotate, which in a significant number of clinical studies has shown stabilization of the disease and an increased progression free survival (PFS), effectiveness in controlling symptoms and an increase in quality of life. In a recent trial, NETTER 1, Lu-DOTA-TATE controlled tumor growth in patients with inoperable, progressive, metastatic, non-functional small intestine NETs. Other large institutional series that include pancreatic NET have revealed high rates of tumor control after PRRT [3,14].

177 Lu-DOTA-TATE has also been described as useful in NETs in other localizations. A cohort study conducted in patients with gastro-entero-pancreatic NETs (GEP NETs), NETs of unknown origin and bronchial tumors revealed that 177Lu-DOTA-TATE treatment determined a progression-free survival of 29 months and an overall survival of 63 months. The response rate of 177Lu-DOTA-TATE treatment was 39%, reaching stabilization of the disease in 43% of patients [15]. The use of PRRT in PPGL has been explored in two small cohorts of patients with inoperable mediastinal or head and neck PPGL, with promising outcomes in terms of tumor response and symptomatic control. The other study was performed in a higher number of patients (*n* = 20). They received four cycles of Lu-DOTATATE with encouraging results in terms of symptomatic control (decreased medication requirements), biochemical control (circulating chromogranin A), tumor response and general control of the disease with a median PFS of 29 months. An ongoing prospective clinical trial at the National Institute of Health (NIH) evaluating PRRT for progressive PPGL will provide definite answers regarding the utility and safety of PRRT in PPGL [16].

**Figure 1:**
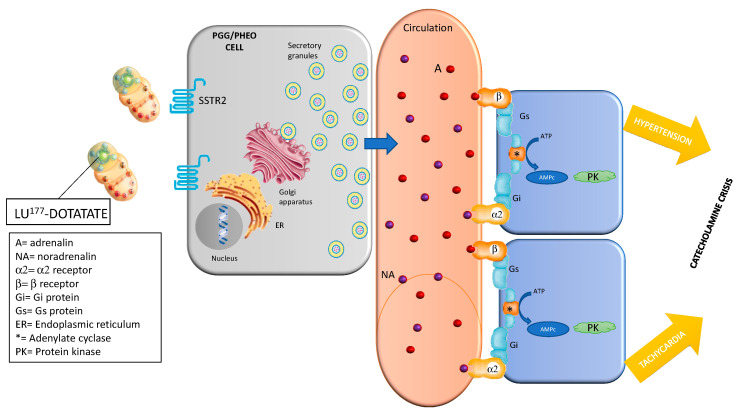
Physiopathology of hormonal crisis during Lu-DOTATATE treatment (catecholamine crisis). Somatostatin receptor 2 (SSTR2); adenosine triphosphate (ATP); cyclic adenosine monophosphate (AMPc); Paraganglioma (PGG); Pheochromocytoma (PHEO).

**Figure 2:**
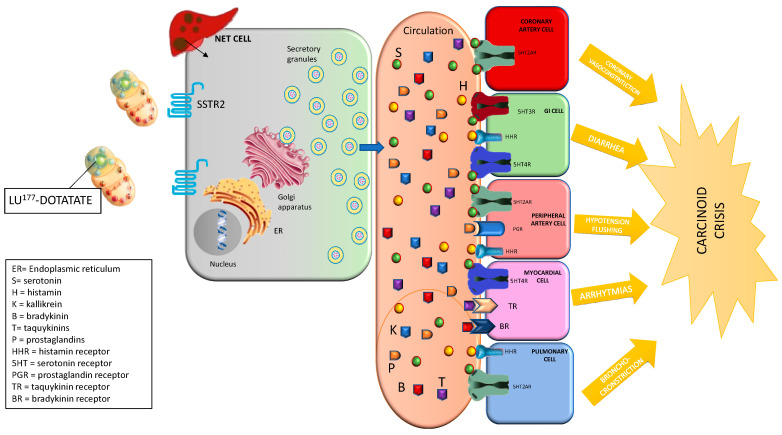
Physiopathology of hormonal crisis during Lu-DOTATATE treatment (carcinoid crisis).

The management of functional NETs before and during LU-DOTA-TATE treatment is specially challenging. Several complications such as severe carcinoid and catecholamine crisis have been described (Figure 1 and Figure 2). The exact mechanism by which these hormonal crises take place during treatment has yet to be elucidated, as there are few reported cases in the literature. Several mechanisms have been postulated, such as SSA discontinuation, emotional stress, amino acid administration during infusion or tumoral lysis due to beta irradiation from 177Lu [17].

The aim of this systematic review is to evaluate hormonal crisis during theragnosis with Lu-DOTA-TATE in functioning neuroendocrine tumors and to establish a practical guidance for the management and prevention of the most common hormonal crisis during radionuclide treatment with Lu-DOTA-TATE: carcinoid syndrome (CS) and catecholamine hypersecretion.

## 2. Methods

**Figure 3:**
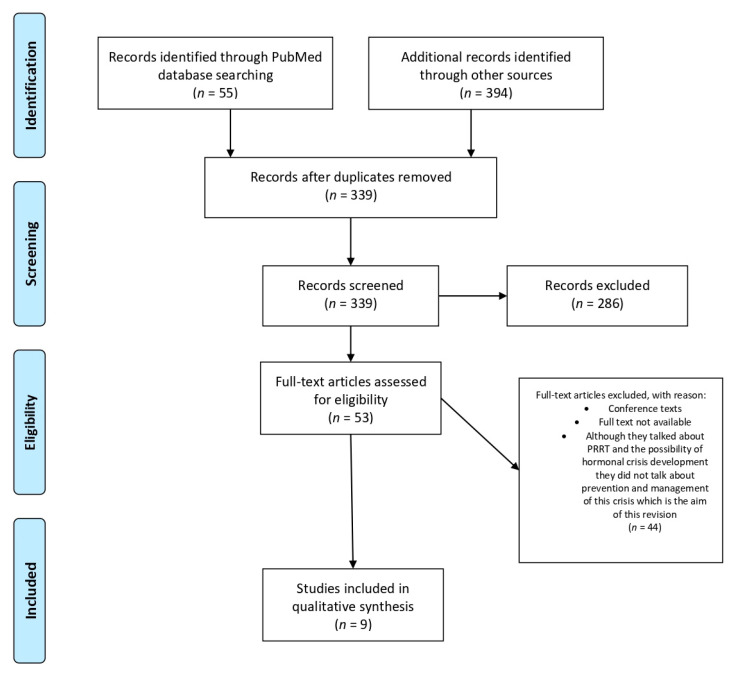
PRISMA flow diagram for studies retrieved through the searching and selection process.

This systematic review was developed according to the Preferred Reporting Items for Systematic Reviews and Meta-Analyses (PRISMA) statement [18] (Figure 3) and conducted using MEDLINE (accessed from PubMed), Google Scholar and ClinicalTrials.gov. The search strategy was based on the Population, Intervention, Comparator, Outcome (PICO) framework [19] and was designed to find studies and reviews including a combination of Medical Subject Headings (MeSH) and non-MeSH keywords related to hormonal crisis during theragnosis with Lu-DOTA-TATE in functioning neuroendocrine tumors: “Peptide Receptor Radionuclide therapy” or “PRRT” and “hormonal crisis”.

As inclusion criteria, we considered that the study sample was human-based, full-text articles and articles available in English. On the other hand, as exclusion criteria, we excluded conference texts, full text not available, and text which, although they talked about PRRT and the possibility of hormonal crisis development, did not talk about prevention and management of this crisis which is the aim of this revision. Two teams consisted of three reviewers in each team (M.I.d.O.-G., J.-F.M.-T., M.A.M., M.L.-d.-l.-T., M.A., P.B.) with expertise in medical and health evaluations and training in research methodology. Any disagreements were resolved by a third researcher (J.M.S.).

According to the National Heart, Lung and Blood Institute [20], the validity of each included study was carried out using nine items to which either the affirmative (+), negative (−) or other, including “cannot determine”, “not applicable” and “not reported”, (that is unclear (?) answers) was allotted and classified in a rating of good (7–9), fair (4–6) or poor (≤ 3) for each individual study.

## 3. Results

For this systematic review, the quality rating of manuscripts published, applying National Heart, Lung and Blood Institute criteria [20], is good. Literature searches located 449 citations potentially addressing all of the key questions of interest for the guideline evidence review. Overall, 53 studies were addressed and considered as evidence, and finally nine were relevant to this systematic review. Of these nine articles only one considered the management of all of the hormonal crisis; the rest either considered the prevention and/or management of carcinoid crisis, hormonal crisis in general excluding catecholaminergic crisis, catecholaminergic crisis or other hormonal complications. Two of the articles included PRRT with MIBG and/or Y90 treatment and management of hormonal crisis and were considered due to the physio-pathological similarity to 177Lu. Table 2 summarizes the prevention and management approach considered in the different articles as well as the hormonal crisis described.

## 4. Discussion

After performing the systematic review, a description of the crisis reported in the literature and an approach proposal for the most frequent hormonal crisis described during PRRT with 177Lu-DOTATE will be described, i.e., carcinoid crisis and catecholaminergic crisis, as well as less frequent metabolic and hormonal complications.

### 4.1. Carcinoid Crisis

CS constitutes the clinical spectrum derived from the secretion of several bioactive amines from NETs and is the most frequent of the hormonal syndromes associated with NETs. CS usually appears in NETs originated in midgut (small intestine, appendix, right colon), and much more unfrequently in NETs originated in foregut (respiratory system, thymus, stomach, duodenum, pancreas). The reported incidence of CS is about 10% of patients with NETs [28]. Carcinoid crisis is a severe complication of CS, in which a massive release of biologically active substances takes place. It is a life-threatening episode manifested by a severe combination of CS symptoms, associated with hemodynamic instability and frequently tachycardia, arrhythmias, metabolic acidosis, and/or mental status disorders, along with a high mortality rate. Carcinoid heart disease is frequent in cases of CS and may add severity in the course of a carcinoid crisis [29]. The criteria for its definition are not well established, so its real incidence is unknown. The most relevant triggers are invasive procedures (e.g., surgery, invasive diagnostic or therapeutic techniques, and/or some medical treatments), including PRRT. According to the literature, incidence during PRRT would be between 1-10% [17,26,27,28,29,30,31,32,33]. This usually takes place during the first Lu-DOTA-TATE cycle, either during the infusion or 12–48 h post-administration [27,29]. Authors agree on the importance of detecting individuals at high risk of developing carcinoid crisis before PRRT. Risk factors are previous CS, elevated 5-hydroxy-indolacetic acid (5-HIAA) and/or Chromogranin A, high tumor burden, metastatic disease (mainly hepatic), carcinoid heart disease, advanced patient age, and the use of drugs that cause histamine release such as sympathomimetic and β2 agonist bronchodilators [26,27,28,29,31,32]. 5HIAA (5-hydroxy-indolacetic acid) determination in urine, a metabolite of 5-HT, constitutes the most useful biomarker in CS cases, or in patients in which prevention of a hormonal crisis might be of paramount importance in metastatic NETs which are going to undergo interventions that might predispose to a severe crisis, such as in PRRT [27]. Its determination requires excluding foods or drugs that interfere. A special diet which excludes certain foods (banana, pineapple, tomato, plums, kiwi, eggplant, avocado) must be initiated 3–5 days prior to its collection.

#### 4.1.1. Prevention

In order to prevent carcinoid crisis, it is advisable to control CS as much as possible by optimizing medical treatment and the patient′s nutritional status, and reducing tumor burden by debulking with surgical resection, radiofrequency ablation, selective internal radiotherapy, or embolization if possible. Nutritional status should be routinely assessed. and malnutrition should be corrected (nicotinamine, niacin, B12, pancreatic enzymes, fat-soluble vitamins supplementation, etc.). Correcting dehydration, electrolyte disorders, and hypoproteinemia are especially important, as well as avoiding alcohol, spicy food, and tryptophan rich food. Physical exertion may also exacerbate the symptoms of CS, so it should be avoided in the days prior to the administration of Lu-DOTA-TATE [24,27,31,32,34]. In functional NETs, and specifically if they present CS, the first-line pharmacological treatment are SSAs. The most frequently used are the i.m. octreotide long-acting release (LAR), at a dose of 10–30 mg every 28 days, or s.c. lanreotide autogel, at a dose of 60–120 mg every 28 days. SSA doses must be adapted to the patients’ symptomatology, which may worsen with disease progression or tachyphylaxis. The short-acting analogue s.c. octreotide may be administered at a dose of 50–200 mcg once to three times a day. Initially it can be used as a test for the tolerability, or later on as a rescue injection when the patient is exhibiting uncontrolled symptomatology. In resistant patients a dose escalation may be needed by shortening the injection interval or increasing dosage, or switching to the other SSA alternative may be considered [34,35,36,37]. To avoid interference with Lu-DOTA-TATE administration in PRRT, SSA long-acting release should not be administered in the previous 28 days and short-acting analog s.c. octreotide in the previous 48 h. However, in patients at high risk of carcinoid crisis, octreotide 100 mcg could be administered before infusion and posteriorly a dose of 50 mcg/h/iv could be maintained [27].

If the patient suffers from a severe CS, other medications may be used to control symptomatology. Diarrhea should be improved with standard diet and antidiarrheal advice, adapting the dose to the symptoms (Loperamide 4 mg initially, with maximum doses 16 mg/day, Codeine 10 mg, with maximum doses 30 mg/6 h) [38]. Cyproheptadine, a serotonin receptor blocker, can be used in doses of 4 mg/8 h. Telotristat ethyl has been found to reduce serotonin production by inhibiting tryptophan hydroxylase (TPH). It is indicated in combination with SSA therapy in adults with CS diarrhea inadequately controlled by SSA therapy [17,24,29,32]. Premedication with benzodiazepines and antihistamines can be useful to reduce anxiety before treatment with PRRT, but its administration is controversial, since histamine release may occur, especially with gastric or bronchial carcinoids [29]. Although steroids may not prevent anaphylactic shock, they can reduce episodes caused by nonspecific histamine release.

#### 4.1.2. Management and Treatment

If CS symptoms appear during the Lu177-DOTATE infusion, the aim at that moment is to block hormonal secretion and the effect of the released mediators in order to control symptomatology (Table 3).

### 4.2. Catecholaminergic Crisis

A high percentage of patients with metastatic paraganglioma and pheochromocytoma express somatostatin receptors, especially SSTR2, which can be used for PRRT. Hormonal crises have been described due to catecholamine secretion in patients with metastatic paraganglioma or pheochromocytoma which can lead to severe hypertension, hypotension, pulmonary edema, myocardial ischemia and shock. These episodes of sudden release of hormones can be induced by multiple factors included physical activity, abdominal pressure, postural changes, anxiety, or smoking. Certain drinks or foods with a high concentration of tyramine (wine, cheese, beers) or drugs (tricyclic antidepressants, histamine, phenothiazine, glucocorticoids, high osmolality ionic contrast agents) and surgical procedures with or without anesthesia can also lead to this release [4].

Several cases have been reported of 131-MIBG treatment in which patients developed hypertension crisis during infusion and even myocardial infarction several days after treatment [39]. However, 177-LUDOTA-TATE treatment-induced catecholamine crises have been barely described. One of the cases described was of a malignant pheochromocytoma in which the patient developed hypotension, sweating and cardiac ischemia 24 h after infusion [17]. In this case, the patient was pretreated with glucocorticoids which may induce catecholamine crisis, as recently described in the literature [40]. Other centers have reported, 24 h following 177-LUDOTATE infusion, severe tumoral lysis syndrome which led to severe hypertension, shortness of breath, tachycardia, hyperkalemia, hyperphosphatemia and hypocalcemia with necessary admission to ICU [22]. Other patients developed severe hypotension or severe tachycardia during treatment infusion [16,17,21,22,25,26,41].

The scientific community appears to be sensitized to the fact that pharmacological treatment of pheochromocytoma and functional paraganglioma before surgery is of vital importance in these patients. However, several authors agree that metastatic functional paraganglioma and pheochromocytoma, which are candidates for PRRT, need to be pretreated and certain protocols should be assessed during treatment infusion in order to avoid the hormonal crisis previously described [16,17,21,25,41,42].

#### 4.2.1. Prevention

In order to control hypertension or hemodynamic stability during radioisotope treatment, patients should undergo pharmacological treatment during the previous days. The aim of this treatment is to block the effects of released catecholamines during infusion. This preoperative therapy is aimed at normalization of blood pressure, heart rate and functioning of other organs; restoration of volume depletion; and prevention of PRRT induced catecholamine storm and its consequences on the cardiovascular system. Nevertheless, international differences in available or approved therapies and a scarcity of evidence-based studies comparing different therapies has led to a lack of consensus [42,43,44,45,46,47].

**Figure 4:**
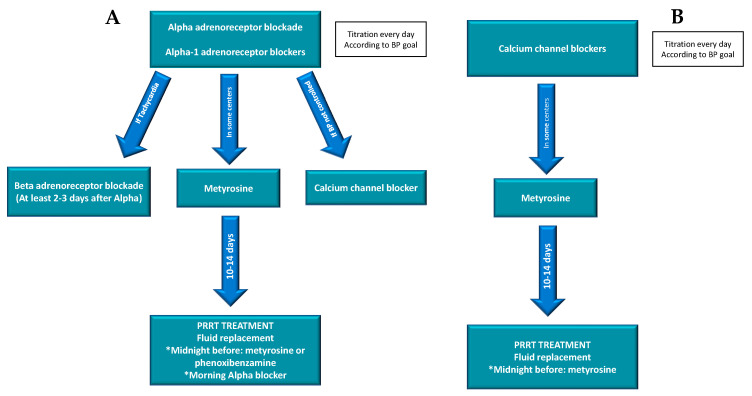
Accepted regimens for preparation of catecholamine producing tumors. (**A**): regimen performed with alpha blockade; (**B**): regimen performed with calcium channel blockers. * specific management for the previous day and day of PRRT: Blood pressure (BP).

Currently there are different medical approaches for patients with catecholamine-producing neoplasms [42,43]. The accepted regimens are combined alpha and beta-adrenergic blockade, calcium channel blockers and metyrosine (tyrosine hydroxylase inhibition) (Figure 4).

In our centers we preferably use the combined alpha and beta-adrenergic blockade. Alpha adrenergic blockade is administrated 10–14 days before treatment to normalize blood pressure and heart rate and expand the contracted blood volume due to the catecholamine secretion. If the patient has organ damage due to long-standing catecholamine excess, this period of alpha-blockade should be longer. Beta-blockers should never be used before the initiation of alpha-blockade in patients with functioning tumors as the unopposed alpha adrenergic effect can cause severe vasoconstriction which can lead to acute cardiac insufficiency, hypertensive crisis, and pulmonary edema. Added therapy with beta-receptor antagonists is required to counteract tachycardia induced by alpha-blocking agents. This tachycardia is a desired side-effect indicating that complete alpha blockade has been achieved. At this point, beta blockers can be added to reduce tachycardia. They must be added to alpha blockade not earlier than 2–3 days later.

With the patient adequately prepared in the days before radioisotope treatment, several precautions should be considered during radioisotope infusion.

Frequently, patients during treatment with 177 LU-DOTA-TATE are pretreated with glucocorticoids infusion; however, this pre-treatment is concerning as catecholamine crisis induced by exogenous glucocorticoids has been described recently. Thus, pre-treatment with glucocorticoids should be avoided [16,17,22,41].

On the other hand, there are several protocols of how to perform radioisotope infusion, and it seems that 177-LUDOTA-TATE infusion should be administered over 2 h at least and preferably over 4 h and not in 15–30 min as described in the literature. During infusion the patient should be monitored closely and intensive care support should be available if needed [22,41].

#### 4.2.2. Management and Treatment

Medical stabilization for pheochromocytoma/paraganglioma crisis requires an individualized approach depending on clinical presentation. The severity of presentation can vary with regard to both hemodynamic stability and extent of organ dysfunction. There is therefore a clinical spectrum, but currently no accepted classification system for this hormonal crisis. In order to control this, general measures and specific treatments can be applied (Table 4) [17,22,43,44,45,46,47].

### 4.3. Other Complications

Other complications that may appear, although less severe than those previously described, but that must be taken into consideration in all patients are glycemic and electrolytic disorders [27,48]. Regarding glycemic disorders it is important to distinguish if the patient has a previous known diabetes or not (Table 5).

If Diabetes Mellitus (DM) is present, record previous antidiabetic treatments and dose, time of last dose, self-glucose monitoring during the previous week, and fasting blood glucose on the day of administration to adjust the treatment on the day of infusion. Likewise, it is important to record medications with hyperglycemic potential, both in patients with DM as well as in those not known to be DM.

If hyperglycemia is present, glucose ≥ 180 mg/dL, it will be treated according to the protocols of glycemic control during hospital stay. Generally, guidelines recommend adjustment with basal insulin, rapid insulin and additional corrections [49].

Hypoglycemia may appear not only in a DM patient but also if the patient has a metastatic insulinoma. Hypoglycemia will be defined according to the American Diabetes Association guidelines [50,51]: mild hypoglycemia (glycemia 56–70 mg/dL), normally with mild symptoms, and severe hypoglycemia (glycemia less or equal that 55 mg/dl), normally with neurological symptoms (Table 6). Management of hypoglycemia will always be intravenous and will differ according to its severity. If mild hypoglycemia, normally a glucose 10% infusion should be enough; if severe hypoglycemia a glucose 30–50% infusion may be needed and, in this case, it is recommended to stop the Lu-DOTA-TATE infusion and to restart it once it has been recovered.

If the patient treated is a metastatic insulinoma and an uncontrolled hypoglycemia takes place, other medications may need to be added before hospital discharge. Usually pharmacological treatments to control hypoglycemia within metastatic insulinoma include diazoxide or SSAs cautiously.

Hypocalcemia is another dreaded complication. It is important to monitor hypocalcemia symptoms and to act accordingly if an episode of tetany takes place. Before Lu-DOTA-TATE, calcium level and corrected calcium level with albumin or total proteins must be assessed. If corrected calcium level is below 8 mg/dL or hypocalcemia symptoms are present, an intravenous treatment with calcium should be started. Usually, a calcium gluconate infusion in 50 mL of 5% dextrose or normal saline infused over 10 to 20 min is used (dosage equivalent to 90 or 180 mg elemental calcium). This dose of calcium gluconate will raise the serum calcium concentration for only two or three hours. If hypocalcemia persists, a slow infusion of calcium containing 1 mg/mL of elemental calcium with normal saline or 5% dextrose water to provide a final volume of 500 mL should be added. Patients typically require 0.5–1.5 mg/kg of elemental calcium per h starting at an initial infusion rate of 50 mL/h (equivalent to 50 mg elemental/h). The dose should be adjusted to maintain the corrected calcium level concentration at the lower end of the normal range. For patients with hypoparathyroidism, calcitriol (oral dose of 0.25–0.5 mcg twice daily) will be introduced and oral calcium (2–3 gr/d) should be initiated as soon as possible. In some cases, it will be necessary to add oral or intravenous magnesium according to response, magnesium levels and kidney function.

Another infrequent complication is that related to tumoral lysis syndrome. This has been described after treatment, usually on the third day, in which uric acid, creatinine and urea increase, with a decreased glomerular filtration rate. Furthermore, hyperkalemia and an occasionally increase of phosphate and calcium levels may also be present. Tumoral lysis after treatment produces a deposit of uric acid and calcium phosphate crystals in the renal tubules with consequent deterioration of kidney function. It is an uncommon complication, but it is important to keep it in mind due to its appearance 2–4 days after treatment [22,52]. Treatment prevention is essential, with adequate hydration and analysis that includes electrolytes, uric acid and kidney function. If it takes place, treatment will be symptomatic.

## 5. Conclusions

The favorable results obtained with PRRT for both NETs and paragangliomas foresee an increase in their indication and even a repositioning in earlier stages of the disease. Studies such as NETTER 2 [53], Study to Evaluate the Efficacy and Safety of Lutathera in Patients with G2 and G3 advanced pancreatic NETs, will answer these questions, among others. The possible adverse effects of PRRT treatments, and especially those of functioning NETs and paragangliomas, require adequate monitoring and treatment given their potential severity.

Functioning NETs and their possible complications should be treated in reference centers with an experienced multidisciplinary team in which each team member contributes with a specialized part of the care plan to optimize treatment with PRRT, as identification of high-risk individuals which are prone to developing hormonal crisis is essential

Further research is needed to establish the optimal 177Lu- DOTA-TATE protocols for treating NETs and paraganglioma in order to minimize severe adverse reactions. Treatment protocols could be modified by lengthening the infusion time of 177Lu-DOTA-TATE and/or lowering the initial treatment dose to help prevent adverse reactions or reduce their severity.

## Figures and Tables

**Table 1:** SSTR expression in NETs according to WHO classification and primary site. + + +, SSTR expression 80–100%; + +, SSTR expression 30–80%; +, SSTR expression 15–30%; −, SSTR expression < 15%.

**WHO Classification**	**SSTR1**	**SSTR2**	**SSTR3**	**SSTR4**	**SSTR5**
G1	+ + +	+ + +	+ +	−	+ + +
G2	+ + +	+ + +	+ +	−	+ +
G3	+ + +	+ + +	+ +	−	−
**Primary Site**	**SSTR1**	**SSTR2**	**SSTR3**	**SSTR4**	**SSTR5**
Pancreas	−	+ + +	−	−	+ +
Insulinoma	+	+ +	+ +	−	+ +
Gastrinoma	+	+ + +	+ +	−	+ +
Gastric	−	+ + +	−	−	+ +
Intestinal	−	+ +	−	−	+ +
Pulmonary					
Typical carcinoma	+ +	+ + +	+ +	−	−
Atypical carcinoma	+ +	+ +	+	−	−
Large cell	+ +	+ +	+ +	−	+
Small cell	+	+ +	+	−	−
Pheochromocytoma	+ + +	+ + +	+ +	−	+ +
Paraganglioma	+ + +	+ + +	+ +	−	+ +

Somatostatin receptors (SSTR); neuroendocrine tumors (NETs); World Health Organization (WHO).

**Table 2:** Articles selected in this systematic review.

Type	Hormonal Crisis Described	Prevention Recommendations	Management Recommendations	Ref.
Retrospective review of efficacy of PRRT on metastatic PHEO and PGG in 20 patients	1 catecholamine crisis (*n* = 20)	Adequate preparation with alpha- and beta-blockade is mandatory, with inpatient monitoring and access to intensive care support if required. Withholding dexamethasone as a premedication should also be considered because this may exacerbate hypertension in patients with PGG/PHEO	nr	[16]
Original article of a retrospective series of 479 patients treated with Lu-DOTATATE during 2000–2007	7 patients/479 1% incidence Carcinoid crisis VIPoma crisis catecholaminergic crisis (*n* = 479) Proposes physio-pathological explanation of these crisis	Alpha and beta blockade in metastatic pheochromocytoma Continuation of SSAs in risk patients Consider withholding of corticosteroids in PHEO/PGG	Fluids Octreotide Loperamide Metoclopramide Corticosteroids Potassium Bisoprolol (in PHEO)	[17]
Multicenter open-label single-arm trial phase II trial to establish efficacy and safety of MIBG in pheochromocytoma and paraganglioma	0 hormonal crisis (*n* = 74)	Strict blood pressure control before treatment. No changes on antihypertensive drugs 30 days before treatment	nr	[21]
Case report and review of 3 paraganglioma and pheochromocytoma patients treated with LU-DOTA-TATE	2/3 hormonal crisis Catecholaminergic crisis 1 required intensive care unit stay	Avoid corticosteroids during infusion Recommendation on lengthening or reduction of dose infusion of Lu-DOTA-TATE	Review on basic treatment of tumoral lysis syndrome and other electrolytic disturbances	[22]
Case report and review of the literature	Case report of carcinoid crisis after Lu-DOTATATE	nr	Treatment of a carcinoid crisis aims at preventing the release of the mediators from tumor tissue and/or blocking their effects on target organs. Intravenous administration of octreotide, corticosteroids, and correction of fluids, and electrolyte disturbances is the backbone of therapy	[23]
Case report	Case report of a carcinoid crisis triggered by PRRT. Death of the patient is reported.	(Pre)treatment with octreotide is recommended for therapeutic interventions in functional midgut NET. Other drugs have been successfully used, cyproheptadine, ketanserin, 5-HT receptor antagonists, corticosteroids, and H1-and H2-receptor antagonist. Somatostatin analogues are considered most effective and are recommended as first-line therapy	Treatment with nimodipine applied in the early phase of the ICU course seemed to be more effective compared to phentolamine. Intravenous octreotide applied in a dose of 500 μg/24 h continuously iv was also ineffective (recommended dose for carcinoid crisis 50–600 μg/day iv).	[24]
Phase II trial of efficacy of PRRT with Y90-DOTATATE in Patients with Advanced, Nonresectable Paraganglioma-Pheochromocytoma, Related to SDHx Gene Mutation 13 patients	No reported hormonal crisis	One of the mainstays to increase the safety of PRRT is that all patients with a hormonally functional PPGL should undergo a pretreatment blockade to prevent cardiovascular complications with alpha-adrenergic receptor blockers as the first choice. Clinicians should avoid medications that can trigger hemodynamic instability and cardiovascular events (for example, steroids, dopamine D2 receptor antagonists, sympathomimetics, selective serotonin reuptake inhibitors, opioid analgesics, tricyclic antidepressants, and others).	nr	[25]
Retrospective study on 504 patients treated with Lu-DOTA-TATE	6 patients with hormonal crisis which required hospitalization	With adequate clinical scrutiny, patients who have an increased risk to develop hormone related crises can be identified and adequate measures to contain such events can be taken. Does not specify which.	nr	[26]
Literature review and cases series from two tertiary hospitals of carcinoid crisis after LU-DOTA-TATE	Seven cases of carcinoid crisis after PRRT	Identification of high-risk cases Correction of electrolyte disturbance, dehydration and hypoproteinemia before PRRT PRRT pre-medication	In the event of carcinoid crisis: octreotide in bolus or continuous infusion. H1 receptor blockers, H2 blockers, and occasionally, steroids.	[27]

nr: not reflected in the study; paragangliomas and pheochromocytomas (PPG/PHEO); Peptide Receptor Radionuclide therapy (PRRT); metaiodobenzylguanidine (MIBG); Neuroendocrine tumor (NET); 5-hydroxytryptamine receptors (5-HT); histamine receptors (H1, H2); intensive care unit (ICU); vasoactive intestinal peptide (VIP).

**Table 3:** General measures for prevention and management of carcinoid crisis.

BEFORE LU-DOTATE INFUSION		
Identify Risk Factors for Carcinoid Crisis	Previous CS, Elevated 5HIAA, Chromogranin A, High Tumor Burden, Metastatic Disease (Mainly Hepatic), Carcinoid Heart Disease, Advanced Age, Histamine Secretion.	
Nutritional assessment [27]	Diagnose and correct hydro-electrolytic disorders	ACTION
		Check sodium, potassium, magnesium, phosphorus levels
	Diagnose and correct malnutrition	Add vitamins and/or supplements
	Diagnose and correct malabsorption	Add pancreatic enzymes
	Avoid food triggers Avoid high intensity exercise the previous days	Recommend diet free of alcohol, spices, or foods rich in tryptophan
Carcinoid tumor [23,26,27]	Tumor debulking	ACTION
		Consider surgery, ablation, radiotherapy, or embolization
	Somatostatin analogs	Octreotide LAR 10–30 mg/28 days Lanreotide autogel 60–120 mg/28 days
	Other antitumoral treatments	
Diarrhea [23,26,27]	Antidiarrheal drugs	ACTION
		Loperamide 4–16 mg/day/oral Codeine 10–90 mg/day/oral
	Anti-serotoninergic drugs Serotonin inhibitors	Cyproheptadine 4 mg/8 h Telotristat ethyl 250 mg/8 h/oral
	Etiopathogenic	Bile acid binders Antibiotics Pancreatic enzymes Niacin supplementation
**DURING LUDOTATATE INFUSION**		
Premedication [21,22,25,30,33,36]	Corticoid treatment	ACTION
		Dexametasone 4–8 mg if high risk patient
	Antiemetic	Ondansetron 4 mg oral
	Somatostatin analogue	Octreotide 100 mcg sc or 50 mcg/iv if high risk patient
	Antihistaminic H1	Dexchlorpheniramine 5 mg iv in slow infusion if high risk patient
	Antihistaminic H2	Ranitidine 50 mg iv in slow infusion if high risk patient
Carcinoid crisis [17,21,26,27,28,30,34,36,37]	Symptomatology control Monitor BP, HR, EKG Maintain volemia	ACTION
		Stop Lu-DOTATATE infusion Octreotide 100–500 mcg sc or iv in saline, maintaining 50–100 mcg/h infusion Consider corticoid treatment (100 mg hydrocortisone or metilprednisolone1–2 mg/kg/iv slow infusion) Consider ICU If hypotension: phenylephrine or vasopresin (in ICU) If hypertension: α, β blockers Saline 0.9% infusion
Flushing, pruritus, uvula or facial edema [25,28,36]	Antihistaminic H1	ACTION
		Dexchlorpheniramine 5–10 mg iv slow infusion, maintain 5 mg/6 h
	Antihistaminic H2	Ranitidine 50 mg iv slow infusion, maintain 50 mg/6–8 h slow infusion
Diarrhea [17,28,30,37]	Monitor BP, HR, EKG	
	Monitor electrolytes	Correct hydro-electrolytic disorders
	Monitor kidney function	Creatinine levels
	Monitor liver function	Hemostasia and transaminases
Bronchospasm [37]	Avoid beta adrenergic stimuli	ACTION
		Avoid terbutalin, salbutamol, salmeterol, bambuterol, indacaterol, olodaterol, formoterol, salmeterol
	Corticoid treatment	Hydrocortisone 100 mg iv slow infusion Metilrednisolone 1–2 mg/kg/iv slow infusion Beclomethasone 100–500 mcg inh Budesonide 200–400 mcg inh Fluticasone 100–250 mcg inh
	Anticholinergic	Bromure ipratropium 50–60 mg inh

5-hydroxy-indolacetic acid (5-HIAA); carcinoid syndrome (CS); intensive care unit (ICU); blood pressure (BP); heart rate (HR); electrocardiogram (EKG).

**Table 4:** General measures for prevention and management of catecholamine crisis.

BEFORE LU-DOTATE INFUSION			
**Identify Risk Factors for Catecholamine Crisis**	Tumors larger than 3–4 cm, uncontrolled blood pressure, high catecholamine levels, or pretreatment orthostatic hypotension		
Nutritional assessment [27,42]	Diagnose and correct hydro-electrolytic disorders Diagnose and correct malnutrition Diagnose and correct constipation Avoid high intensity exercise the previous days	ACTION	
		Check sodium, potassium, magnesium, phosphorus levels Add vitamins and/or supplements Specific diet for constipation	
Catecholamine producing tumor [32,45]	Tumor debulking	ACTION	
		Consider surgery, ablation, radiotherapy, or embolization	
	Alpha adrenergic blockade	Phenoxybenzamine	Initial dose: 10 mg 1–2 times day Titration: 10–20 mg every 2–3 days Average daily dose: 20–100 mg/day Maximum dose: 240 mg/day
		Prazosin	Initial dose: 0.5–1 mg per dose every 4–6 h Average daily dose: 2–5 mg two or three times a day Maximum dose: 20–24 mg/day
		Doxazosin	Initial dose: 1–2 mg/day TitrationMaximum dose: 16 mg/day
		Terazosin	Initial dose: 1 mg/day Titration Average dose: 2–5 mg/day Maximum dose: 20 mg/day
	Beta adrenergic blockade	Metoprolol	25–50 mg three to four times a day
		Atenolol	12.5–25 mg two to three times a day
		Propranolol	20–80 mg one to three times a day
	Metyrosine	Initial dose: 250 mg orally every 8–12 h Titration Average dose: 1.5–2 gr per day High fluid intake to avoid crystalluria is suggested for patients taking more than 2 gr/day	
	Calcium channel blockers	Amlodipine	10–20 mg/day
		Nicardipine	60–90 mg/day
		Verapamil	180–540 mg/day
**DURING LUDOTATATE INFUSION**			
Premedication [16,17,24,29]	Corticoid treatment	ACTION	
		AVOID	
	Antiemetic	Ondansetron 4 mg oral	
Catecholamine crisis [24,29,45,46,47,48,49]	Symptomatology control	Stop Lu-DOTATATE infusion Consider slowing infusion rate over 2 h at least and preferably during 4 h	
	Monitor BP, HR, EKG Maintain volemia	Saline 0.9% infusion	
	If hypertension	Captopril 50 mg oral	
	If severe hypertension	Sodium nitroprusside: 0.5–5.0 mcg/kg /min, Maximum dose 3 mcg/kg/min Phentolamine: initial dose of 1 mg, if necessary, repeat 5 mg boluses or continuous infusion Nicardipine started at 5 mg/h and titrated for blood pressure control (may be increased by 2.5 mg/h every 15 min up to a maximum of 15 mg/h).	
	If hypertension + tachycardia	Labetalol infusion 20 mg/iv in slow boluses every 5–10 min until a maximum dose of 300 mg. If continuous infusion is needed 250 mg in 250 mL of glucose 5% at a rhythm of 2–10 mg/min.	
	If cardiac arrhythmias	Lidocaine 50–100 mg intravenously Esmolol (50–200 mcg/kg/min intravenously)	
	If other complications or not control	Always consider ICU	

Blood pressure (BP); heart rate (HR); electrocardiogram (EKG); histamine receptors (H1, H2); intensive care unit (ICU).

**Table 5:** Glycemic control and treatment in patients undergoing Peptide Receptor Radionuclide therapy (PRRT).

General Recommendations	Before Infusion	Known Diabetes	Unknown Diabetes	After Infusion
Digital blood glucose [49]	Glucose test	Every h	Every two h	Before meals or every 6 h if fasting
Treated with oral drugs [49]	Do not administer oral DM drugs that morning	Correction with rapid insulin if indicated	Correction with rapid insulin if indicated	Restart before dinner if indicated
	If Metformin, withdrawal 48 h before. DPP4 inhibitor, can be used			
Treated with insulin [49]	Administer Basal dose	Adjust with rapid insulin every 6 h		Restart before lunch if indicated
Fluid therapy [49]	Start glucose 5% 500 mL/6 h or 10% 500/12 h			Stop glucose when oral tolerance
If Hypoglycemia [50]	Treatment according general recommendations			

Dipeptidyl peptidase 4 inhibitor (DDP4 inhibitor).

**Table 6:** Hypoglycemia treatment in patients undergoing PRRT.

General Recommendations	Glucose 56–70 mg/dL	Glucose < 55 mg/dL or Neurologic Symptoms
Glucose test [50,51]	Every 15 min up to glucose > 80 mg/dL	Every 5 min up to glucose > 80 mg/dL
Glucose 10% [50,51]	100 mL (glucose 10 g) in 5–10 min. Repeat if necessary.	
Glucose 50% [50,51]		30 mL (Glucose 15 g) bolus. Repeat if necessary.
Fluid [50,51]	Glucose 5–10% continuous, 500 mL/4–12 h Minimum 100 g of glucose in 24 h	
